# Integrative Analysis of *ATP2B4* Expression and Its Clinical and Molecular Associations in Colorectal Cancer

**DOI:** 10.3390/cimb48100977

**Published:** 2026-09-23

**Authors:** Kai Aoki, Mitsugi Shimoda, Kazuhiro Takekoshi

**Affiliations:** 1 Department of Health and Nutrition, Faculty of Health Science, Niigata University of Health and Welfare, Niigata 950-3198, Japan; 2 Department of Gastroenterological Surgery, Ibaraki Medical Center, Tokyo Medical University, Ibaraki 300-0395, Japan; mshimoda@tokyo-med.ac.jp; 3 Institute of Medicine, University of Tsukuba, Ibaraki 305-8576, Japan; k-takemd@md.tsukuba.ac.jp

**Keywords:** colorectal cancer, *ATP2B4*, PMCA4, calcium signaling, bioinformatics

## Abstract

Plasma membrane Ca^2+^-ATPase isoform 4 (PMCA4), encoded by the *ATP2B4* gene, is a key regulator of intracellular Ca^2+^ homeostasis and has recently emerged as a context-dependent modulator of cancer progression. However, its clinicopathological significance in colorectal cancer (CRC) remains incompletely characterized. Here, we performed an integrated bioinformatic analysis using multiple publicly available datasets to evaluate *ATP2B4* expression at the transcript and protein levels, its association with patient survival and clinicopathological characteristics, and its molecular context in CRC. *ATP2B4* expression was additionally evaluated in independent CRC datasets to assess the reproducibility of the observed expression and prognostic associations. Transcriptomic and proteomic analyses demonstrated significantly reduced expression in tumor tissues compared with normal colorectal tissues. Survival analysis using the KM-Plotter dataset showed associations between higher *ATP2B4* expression and poorer overall survival; however, this prognostic association was not reproduced in the independent cohort using a median-based cutoff. Further analyses showed that *ATP2B4* expression was associated with tumor purity, stromal content, and molecular subtype, indicating that bulk-tumor expression patterns are influenced by tumor composition and molecular context. Co-expression and functional enrichment analyses indicated positive associations with cell adhesion and extracellular matrix organization, whereas tumors with low expression were enriched for mitochondrial and oxidative phosphorylation pathways. Collectively, these findings demonstrate reproducible downregulation of *ATP2B4* in CRC while showing that its prognostic and molecular associations are context dependent and should be interpreted in relation to tumor composition and molecular subtype.

## 1. Introduction

Colorectal cancer (CRC) is one of the most commonly diagnosed malignancies worldwide and remains a leading cause of cancer-related mortality despite substantial advances in early detection, surgical techniques, systemic chemotherapy, and molecularly targeted therapies [1]. Although recent molecular classifications have improved patient stratification and therapeutic decision-making, considerable heterogeneity in clinical behavior and prognosis persists even among patients with similar clinicopathological characteristics [2,3]. These observations underscore the importance of identifying molecular factors associated with CRC heterogeneity and clinical outcome while improving our understanding of the biological processes underlying CRC progression.

Intracellular Ca^2+^ functions as a ubiquitous second messenger that regulates diverse cellular processes, including proliferation, apoptosis, migration, differentiation, metabolism, and cell adhesion [4,5,6,7,8,9]. Rather than representing passive consequences of malignant transformation, alterations in Ca^2+^ signaling are now recognized as active contributors to tumor progression. Cancer cells extensively remodel the Ca^2+^ signaling machinery, including ion channels, pumps, exchangers, and intracellular Ca^2+^ stores, to generate Ca^2+^ dynamics that support survival, proliferation, invasion, metastasis, and therapeutic resistance. Consequently, molecules responsible for maintaining intracellular Ca^2+^ homeostasis have emerged as important components of cancer biology and potential targets for further investigation [6,9].

Among the regulators of intracellular Ca^2+^ homeostasis, plasma membrane Ca^2+^-ATPase isoform 4 (PMCA4), encoded by the *ATP2B4* gene, belongs to the plasma membrane Ca^2+^-ATPase family. PMCA4 was originally characterized as a high-affinity, ATP-dependent Ca^2+^ extrusion system that maintains low cytosolic Ca^2+^ concentrations [10,11]. More recent studies have demonstrated that PMCA4 not only functions as a Ca^2+^ extrusion pump but also serves as an organizer of localized signaling complexes. Through interactions with multiple signaling proteins, PMCA4 contributes to spatially restricted Ca^2+^ signaling and thereby influences diverse physiological and pathological processes [12,13,14]. PMCA4 is therefore increasingly recognized as a signaling regulator rather than merely a Ca^2+^ extrusion pump.

In colorectal cancer, previous studies have demonstrated that PMCA4 expression increases during differentiation of colorectal cancer cells and is reduced in human colon tumors [15,16]. Experimental modulation of PMCA4 expression in colon cancer cells has further shown that PMCA4 affects intracellular Ca^2+^ responses and cell proliferation [16]. However, despite these experimental and expression-level observations, the reproducibility of *ATP2B4*/PMCA4 downregulation across independent cohorts and its clinical and molecular associations in CRC remain incompletely characterized. Beyond colorectal cancer, accumulating evidence indicates that the biological role of PMCA4 is highly context dependent. Depending on the tissue type and cellular context, PMCA4 has been reported to exert either tumor-suppressive or tumor-promoting effects. Altered PMCA4 expression has been implicated in cancer cell proliferation, migration, epithelial plasticity, apoptosis, stemness, therapeutic resistance, and metastatic potential in breast [17], pancreatic [18], gastric [19], and melanoma [20,21] cancers. Collectively, these findings highlight the importance of evaluating *ATP2B4*/PMCA4 within the specific molecular and cellular context of each cancer type.

Despite growing interest in PMCA4 biology, an integrated characterization of *ATP2B4* expression and its clinical and molecular associations in CRC has not, to our knowledge, been reported. Therefore, in the present study, we analyzed multiple publicly available transcriptomic, proteomic, survival, and functional genomic datasets to characterize *ATP2B4*/PMCA4 expression and its clinical and molecular associations in CRC. Independent cohorts were incorporated to evaluate the reproducibility of expression and survival findings, and additional analyses accounting for tumor composition and consensus molecular subtype were performed to examine the context of the observed associations. Co-expression and functional enrichment analyses were further used to characterize the transcriptional programs associated with *ATP2B4* expression.

## 2. Materials and Methods

### 2.1. Study Design

This study integrated publicly available transcriptomic, proteomic, and clinical datasets to characterize *ATP2B4* expression and its associations with clinicopathological features, overall survival, tumor composition, molecular subtype, and co-expression-derived biological pathways in CRC. *ATP2B4* mRNA expression was evaluated using The Cancer Genome Atlas colon adenocarcinoma cohort (TCGA-COAD), and PMCA4 protein abundance was evaluated using colorectal cancer proteomic data from the Clinical Proteomic Tumor Analysis Consortium (CPTAC). GSE44076 was used as an independent cohort to validate tumor-associated changes in *ATP2B4* expression. Survival associations were initially explored using KM Plotter and subsequently evaluated in the independent GSE39582 cohort. GSE39582 was additionally used to examine the relationships of *ATP2B4* expression with stromal content, estimated tumor purity, and consensus molecular subtype (CMS), and to perform multivariable survival analyses. Co-expression and functional enrichment analyses were performed using LinkedOmics.

All analyses were based exclusively on publicly available, de-identified datasets. No new human participants were enrolled in this study.

### 2.2. ATP2B4 mRNA and PMCA4 Protein Expression Analyses

*ATP2B4* mRNA expression was evaluated using RNA-sequencing data from TCGA-COAD obtained from the Genomic Data Commons (GDC) (https://portal.gdc.cancer.gov/ (accessed on 15 July 2026, re-accessed between 25 and 31 August 2026)) [22]. *ATP2B4* expression was extracted from the tpm_unstranded field of STAR Counts files and expressed as transcripts per million (TPM). Multiple RNA-sequencing files corresponding to the same sample were averaged, and when multiple samples from the same case and biological group were present, sample-level TPM values were further averaged to obtain one value per case and group. The final dataset included 41 normal tissues and 458 primary tumors. For visualization, *ATP2B4* expression was transformed as log2(TPM + 1), whereas inferential statistical analyses were performed using untransformed TPM values. *ATP2B4* expression was compared between normal tissues and primary tumors using the Wilcoxon rank-sum test. A paired analysis was additionally performed for 41 cases with matched normal and primary tumor tissues using the paired Wilcoxon signed-rank test.

AJCC pathological stage information was retrieved from the GDC Cases application programming interface. Pathological substages were collapsed into stages I–IV. Cases with discordant major pathological stage assignments across multiple diagnostic records were excluded. The final stage-stratified tumor dataset consisted of 75 stage I, 175 stage II, 128 stage III, and 64 stage IV tumors. Differences among normal tissues and the four pathological stages were assessed using the Kruskal–Wallis test. Pairwise comparisons between normal tissues and each pathological stage were performed using Wilcoxon rank-sum tests, with adjustment for the four comparisons using the Holm method. The adjusted pairwise results are provided in Table S1.

PMCA4 protein abundance was evaluated using CPTAC colorectal cancer proteomic data (https://pdc.cancer.gov (accessed on 15 July 2026, re-accessed between 25 and 31 August 2026)) [23]. *ATP2B4* was extracted from the proteomic matrix, and protein abundance was represented by the Unshared Log Ratio. Pooled reference samples were excluded, and proteomic data were integrated with the corresponding biospecimen and clinical metadata. The final dataset included 100 normal tissues and 97 primary tumors. PMCA4 abundance was compared between normal tissues and primary tumors using the Wilcoxon rank-sum test. Matched normal and primary tumor specimens were available for 96 patients and were compared using the paired Wilcoxon signed-rank test. For stage-stratified analyses, pathological substages were collapsed into stages I–IV; one primary tumor without an assignable pathological stage was excluded, yielding 10 stage I, 39 stage II, 40 stage III, and 7 stage IV tumors. Differences among normal tissues and pathological stages were assessed using the Kruskal–Wallis test, followed by normal-versus-stage Wilcoxon rank-sum tests with Holm adjustment for four comparisons. These adjusted comparisons are also provided in Table S1.

As an independent validation of the tumor-associated change in *ATP2B4* expression, the GSE44076 dataset (GPL13667) was obtained from the Gene Expression Omnibus (GEO) using the GEOquery package. Five *ATP2B4*-associated probes were identified from the platform annotation: 11718081_a_at, 11718082_a_at, 11718083_a_at, 11746528_a_at, and 11756038_x_at. Expression was evaluated in matched normal colorectal mucosa and primary colorectal tumor tissues from 98 patients (98 matched pairs). Differences between matched normal and tumor tissues were assessed using paired Wilcoxon signed-rank tests, and *p* values across the five probes were adjusted using the Benjamini–Hochberg method. Expression distributions and raw *p* values are shown in Figure S1, and the corresponding raw and BH-adjusted *p* values are provided in Table S2.

### 2.3. Survival Analysis Using KM Plotter

The association between *ATP2B4*-associated probe expression and overall survival was initially explored using the KM Plotter online survival analysis platform [24]. Three *ATP2B4*-associated Affymetrix probes were evaluated: 212136_at, 212135_s_at, and 205410_s_at. Probe 212136_at is annotated as MXRA1/*ATP2B4* in KM Plotter. For each probe, patients were divided into high- and low-expression groups using the median expression value as the cutoff. The analysis included 1061 patients, with 531 patients in the low-expression group and 530 patients in the high-expression group. Kaplan–Meier curves were generated for overall survival, and hazard ratios with 95% confidence intervals and log-rank *p* values were obtained from KM Plotter. The median cutoff was used throughout; an optimized or best-performing cutoff was not used.

### 2.4. Independent Survival and Tumor Context Analyses in GSE39582

An independent evaluation of the association between *ATP2B4* expression and overall survival was performed using GSE39582, obtained from GEO using the GEOquery package. The ExpressionSet containing 585 samples was selected. Three *ATP2B4*-associated probes corresponding to those examined using KM Plotter were evaluated: 212136_at, 212135_s_at, and 205410_s_at. Probe 212136_at was designated as the primary probe, whereas 212135_s_at and 205410_s_at were evaluated as alternative probes in sensitivity analyses. Overall survival time and event status were obtained from the accompanying clinical annotation. Samples with complete probe-expression and survival information were included. For each probe, patients were dichotomized according to the median expression value, with values equal to the median assigned to the high-expression group. For the primary probe, 579 patients with 194 events were available, comprising 289 patients in the low-expression group and 290 in the high-expression group. Kaplan–Meier survival curves were compared using the log-rank test, and hazard ratios with 95% confidence intervals for high versus low expression were estimated using Cox proportional hazards regression.

To evaluate the relationship between *ATP2B4* expression and tumor composition, Affymetrix probes were mapped to HGNC gene symbols using the hgu133plus2.db annotation package. When multiple probes mapped to the same gene symbol, their expression values were averaged to obtain a single gene-level value. The resulting expression matrix was analyzed using the ESTIMATE algorithm. Common genes were filtered using filterCommonGenes, and StromalScore, ImmuneScore, ESTIMATEScore, and estimated TumorPurity were calculated using estimateScore with the platform specified as affymetrix. Associations between individual *ATP2B4* probe expression and StromalScore or TumorPurity were evaluated using Spearman’s rank correlation. Linear regression lines in the scatterplots were included solely to visualize the direction of the associations and were not used for statistical inference. Analyses using 212136_at are shown in the main figure, whereas corresponding sensitivity analyses using 212135_s_at and 205410_s_at are shown in Figure S2.

Consensus Molecular Subtype (CMS) classification was performed using the CMScaller package and the templates. CMS reference template. The gene-level expression matrix described above was used as input, with a random seed of 12345 set for reproducibility. CMS classification was assigned to 521 tumors: CMS1, *n* = 83; CMS2, *n* = 176; CMS3, *n* = 98; and CMS4, *n* = 164. Differences in *ATP2B4*-associated probe expression among CMS1–CMS4 were evaluated using the Kruskal–Wallis test, followed by Dunn’s post hoc pairwise comparisons with Benjamini–Hochberg adjustment for multiple comparisons. The primary analysis using 212136_at is shown in the main figure, whereas corresponding analyses using 212135_s_at and 205410_s_at are presented in Figure S2. The pairwise comparisons for all three probes are provided in Table S3.

Multivariable Cox proportional hazards models were subsequently constructed to evaluate the association between continuous *ATP2B4*-associated probe expression and overall survival while accounting for clinical characteristics and tumor context. Separate analyses were performed for each of the three probes. Model 1 included continuous probe expression, age, sex, and TNM stage. Model 2 additionally included StromalScore. To examine the incorporation of CMS into the survival model, a diagnostic model additionally included CMS as a conventional covariate. The proportional hazards assumption was assessed using Schoenfeld residuals with the cox.zph function. Because CMS violated the proportional hazards assumption when included as a conventional covariate, CMS was incorporated as a stratification factor in the final model, allowing baseline hazard functions to differ among CMS1–CMS4. The final model therefore included continuous probe expression, age, sex, TNM stage, and StromalScore, with CMS incorporated using strata (CMS). Models 1 and 2 included 574 patients with 193 events, whereas the final CMS-stratified model included 510 patients with 171 events. Hazard ratios, 95% confidence intervals, and *p* values for *ATP2B4*-associated probe expression are presented in Table 1.

### 2.5. LinkedOmics Co-Expression and Functional Enrichment Analyses

*ATP2B4* co-expression analysis was performed using the LinkedOmics database [25]. In LinkFinder, *ATP2B4* was selected as the search attribute in the TCGA_COADREAD RNA-sequencing dataset at the gene level using Firehose_RSEM_log2 expression data. The analysis included 379 patients and 19,828 target attributes. Associations between *ATP2B4* and other gene-expression attributes were evaluated using the Pearson correlation test. Genes were ranked according to their correlations with *ATP2B4*. The overall correlation distribution, the association between *ATP2B4* and TNS1, and heatmaps of the 50 most positively and 50 most negatively correlated genes were visualized. The complete LinkFinder correlation ranking is provided as Supplementary Data S1.

Functional enrichment analyses were performed using the LinkInterpreter module. For over-representation analysis, the Overrepresentation Enrichment Analysis option was selected. Gene Ontology (GO) Biological Process and Kyoto Encyclopedia of Genes and Genomes (KEGG) pathway analyses were performed separately. FDR from the LinkFinder results was selected as the rank criterion, and positively and negatively correlated genes were analyzed separately using a significance level of 0.05.

Gene set enrichment analysis (GSEA) was also performed using LinkInterpreter. GO Biological Process was selected as the enrichment database, FDR from the LinkFinder results was used as the rank criterion with signed-ranked data, the minimum gene-set size was set to 10, and 5000 simulations were performed. Enrichment results with FDR < 0.05 were considered statistically significant.

### 2.6. Statistical Analysis

Statistical analyses of expression and protein abundance were performed using nonparametric methods. Comparisons between two independent groups were performed using the Wilcoxon rank-sum test, paired comparisons using the paired Wilcoxon signed-rank test, and comparisons involving more than two groups using the Kruskal–Wallis test. Where applicable, post hoc pairwise comparisons were performed using Wilcoxon rank-sum tests with Holm adjustment or Dunn’s test with Benjamini–Hochberg adjustment, as specified above. Benjamini–Hochberg adjustment was also applied across the five *ATP2B4*-associated probes in the GSE44076 analysis.

Associations between *ATP2B4* expression and ESTIMATE-derived scores were assessed using Spearman’s rank correlation. The LinkedOmics LinkFinder analysis used Pearson correlation according to the selected analysis settings. Survival distributions were compared using the log-rank test, and Cox proportional hazards regression was used to estimate hazard ratios and 95% confidence intervals. The proportional hazards assumption was evaluated using Schoenfeld residuals. All statistical tests were two-sided, and *p* < 0.05 or adjusted *p* < 0.05, as applicable, was considered statistically significant.

Statistical analyses and figure generation for the TCGA-COAD, CPTAC, GSE44076, and GSE39582 datasets were performed using R version 4.6.1. Detailed R scripts for reproducibility are provided as Supplementary Code.

## 3. Results

### 3.1. ATP2B4 mRNA and PMCA4 Protein Abundance Are Decreased in Colorectal Cancer

*ATP2B4* mRNA expression was evaluated in the TCGA-COAD cohort. *ATP2B4* expression was significantly lower in primary tumors (*n* = 458) than in normal colorectal tissues (*n* = 41) (Figure 1A). This difference was also observed in the paired analysis of 41 patients with matched normal and primary tumor tissues (Figure 1B). *ATP2B4* expression differed significantly among normal tissues and tumors stratified by pathological stage (Kruskal–Wallis test, *p* < 0.001), with significantly lower expression in each tumor stage than in normal tissues after Holm adjustment for multiple comparisons (Figure 1C and Table S1).

CPTAC proteomic analysis similarly showed significantly lower PMCA4 protein abundance in primary tumors (*n* = 97) than in normal tissues (*n* = 100) (Figure 1D). PMCA4 abundance was also significantly lower in primary tumors in the paired analysis of 96 matched normal and tumor specimens (Figure 1E). PMCA4 abundance differed significantly among normal tissues and pathological stages (Kruskal–Wallis test, *p* < 0.001), with significantly lower abundance in each tumor stage than in normal tissues after Holm adjustment (Figure 1F and Table S1).

*ATP2B4* expression was also examined in the independent GSE44076 cohort using five *ATP2B4*-associated probes in 98 matched pairs of normal colorectal mucosa and primary colorectal tumors. Four of the five probes showed significantly lower expression in tumor tissues after Benjamini–Hochberg adjustment, whereas 11718082_a_at showed no significant difference (Figure S1 and Table S2).

### 3.2. ATP2B4-Associated Probes Show Prognostic Associations in KM Plotter but Not in the Independent GSE39582 Cohort

The association between *ATP2B4* expression and overall survival was examined using three *ATP2B4*-associated microarray probes in the KM Plotter cohort (*n* = 1061). Higher expression of 212136_at was significantly associated with poorer overall survival (HR = 1.32, 95% CI: 1.08–1.62; log-rank *p* = 0.0067) (Figure 2A). Higher expression of 212135_s_at was also significantly associated with poorer overall survival (HR = 1.45, 95% CI: 1.18–1.77; *p* = 0.0004) (Figure 2B), whereas the association for 205410_s_at did not reach statistical significance (HR = 1.21, 95% CI: 0.99–1.48; *p* = 0.068) (Figure 2C).

The same three probes were subsequently examined in the independent GSE39582 cohort. Among 579 patients with 194 overall-survival events, median-based Kaplan–Meier analyses showed no significant association between expression of any of the three *ATP2B4*-associated probes and overall survival (Figure 3A–C).

### 3.3. ATP2B4 Expression Is Associated with Tumor Composition and Consensus Molecular Subtype

The relationship between *ATP2B4* expression and tumor composition was examined in GSE39582 using ESTIMATE-derived metrics. Expression of 212136_at was positively correlated with StromalScore and negatively correlated with estimated tumor purity (Figure 4A,B). Corresponding analyses using the alternative *ATP2B4*-associated probes 212135_s_at and 205410_s_at are shown in Figure S2.

Expression of 212136_at differed significantly among CMS1–CMS4 (Kruskal–Wallis test, *p* < 0.001; Figure 4C). Dunn’s post hoc tests with Benjamini–Hochberg adjustment identified significant pairwise differences among the CMS groups (Table S3). Expression of 212135_s_at and 205410_s_at also differed significantly among CMS groups, with probe-specific patterns in the pairwise comparisons (Figure S2 and Table S3).

Multivariable Cox proportional hazards analyses were performed using each *ATP2B4*-associated probe as a continuous variable (Figure 4D and Table 1). In the clinical model adjusted for age, sex, and TNM stage, higher 212135_s_at expression was significantly associated with poorer overall survival (HR = 1.28, 95% CI: 1.06–1.55; *p* = 0.012), whereas 212136_at (HR = 1.16, 95% CI: 0.97–1.38; *p* = 0.099) and 205410_s_at (HR = 1.31, 95% CI: 0.98–1.76; *p* = 0.069) were not significantly associated with overall survival. After inclusion of StromalScore, none of the three probes was significantly associated with overall survival. In the final model incorporating CMS as a stratification factor, none of the three probes was significantly associated with overall survival (212136_at: HR = 1.09, 95% CI: 0.84–1.40, *p* = 0.517; 212135_s_at: HR = 1.16, 95% CI: 0.88–1.54, *p* = 0.300; 205410_s_at: HR = 1.14, 95% CI: 0.80–1.63, *p* = 0.457) (Figure 4D and Table 1).

### 3.4. ATP2B4 Expression Is Associated with Distinct Co-Expression Patterns in Colorectal Cancer

Genome-wide co-expression analysis was performed using LinkedOmics in the TCGA COADREAD cohort (*n* = 379). *ATP2B4* expression was positively and negatively correlated with multiple genes across the transcriptome (Figure 5A). The complete LinkFinder results are provided in Supplementary Data S1.

Among the positively correlated genes, TNS1 showed a strong positive correlation with *ATP2B4* expression (Pearson’s r = 0.8245, *p* < 0.0001; Figure 5B). Heatmaps of the top 50 genes positively and negatively correlated with *ATP2B4* expression are shown in Figure 5C and Figure 5D, respectively.

### 3.5. Functional Enrichment Analysis of ATP2B4-Associated Genes

Functional enrichment analyses were performed for genes associated with *ATP2B4* expression. GO Biological Process analysis identified significantly enriched terms among genes positively and negatively correlated with *ATP2B4* (Figure 6A,B). KEGG pathway analysis similarly identified significantly enriched pathways among positively and negatively correlated genes (Figure 6C,D).

GSEA identified gene sets positively and negatively associated with *ATP2B4* expression (Figure 6E). Integrin-mediated signaling was positively associated with *ATP2B4* expression, whereas NADH dehydrogenase complex assembly was negatively associated with *ATP2B4* expression (Figure 6F,G).

## 4. Discussion

PMCA4, encoded by *ATP2B4*, has traditionally been recognized as a plasma membrane Ca^2+^ extrusion pump. However, accumulating evidence indicates that PMCA4 has broader roles in localized Ca^2+^ signaling and exhibits context-dependent functions across different tissues and disease states [26]. To clarify the clinical and molecular associations of *ATP2B4*/PMCA4 in colorectal cancer, we performed an integrated bioinformatic analysis using multiple publicly available transcriptomic, proteomic, survival, and functional genomic datasets. In addition, independent cohorts and analyses accounting for tumor composition and molecular subtype were incorporated to evaluate the robustness and clinical context of the observed associations.

Our integrated analyses consistently demonstrated that *ATP2B4*/PMCA4 expression was reduced at both the transcriptomic and proteomic levels in colorectal tumors. *ATP2B4* mRNA expression was significantly lower in tumors than in normal colorectal tissues in the TCGA-COAD cohort, and this reduction was observed across pathological stages. Consistent with the transcriptomic findings, CPTAC analysis demonstrated reduced PMCA4 protein abundance in colorectal tumors. Importantly, reduced *ATP2B4* expression was independently reproduced in the GSE44076 cohort, in which four of five *ATP2B4*-associated probes showed significantly lower expression in matched tumor tissues. These findings are consistent with a previous study demonstrating reduced PMCA4 expression in human colon tumors and colon adenocarcinomas [16]. Experimental studies in human colon cancer cells have further shown that modulation of PMCA4 expression alters intracellular Ca^2+^ homeostasis and cell proliferation [16], providing a biological basis for considering changes in PMCA4 expression in relation to colorectal cancer cell phenotypes. Thus, the present findings extend previous observations by demonstrating that reduced *ATP2B4*/PMCA4 expression is reproducibly observed across independent transcriptomic and proteomic datasets. More broadly, intracellular Ca^2+^ signaling is governed by coordinated interactions among Ca^2+^ channels, pumps, intracellular stores, and downstream signaling pathways, and remodeling of this network is increasingly recognized as an important feature of cancer biology [6,9,27]. Within this broader context, altered *ATP2B4*/PMCA4 expression may represent one component of the Ca^2+^-signaling changes associated with CRC. However, whether reduced PMCA4 expression contributes directly to colorectal tumorigenesis, how it affects Ca^2+^ signaling in colorectal tumors, and how it interacts with other components of the Ca^2+^-signaling network remain to be determined.

Although higher expression of two *ATP2B4*-associated probes was significantly associated with poorer overall survival in the KM Plotter cohort, these associations were not reproduced in the independent GSE39582 cohort using median-based Kaplan–Meier analysis. Furthermore, multivariable analyses in GSE39582 provided limited evidence for an independent prognostic association. Although higher expression of 212135_s_at remained significantly associated with poorer overall survival after adjustment for age, sex, and TNM stage, this association was no longer significant after additional adjustment for StromalScore, and none of the three probes was significantly associated with overall survival when CMS was incorporated into the model. These findings indicate that the prognostic association of *ATP2B4* is not consistent across cohorts and may be influenced by clinicopathological and molecular characteristics of the tumors. Therefore, the present results do not support *ATP2B4* expression alone as an independent prognostic biomarker for colorectal cancer.

The relationship between *ATP2B4* expression and tumor composition provides an important context for interpreting these prognostic findings. In GSE39582, *ATP2B4* expression was positively associated with StromalScore and negatively associated with estimated tumor purity, indicating that *ATP2B4* expression measured in bulk tumor specimens may partly reflect differences in cellular composition rather than malignant epithelial cells alone. *ATP2B4* expression also differed significantly among consensus molecular subtypes, further demonstrating its association with the molecular heterogeneity of colorectal cancer. This is particularly relevant because the CMS classification captures distinct biological and microenvironmental characteristics, with CMS4 tumors characterized by prominent stromal infiltration and an unfavorable clinical course [2]. Accordingly, the attenuation of the prognostic association after accounting for StromalScore and CMS is consistent with the possibility that tumor composition and molecular subtype contribute to the apparent relationship between *ATP2B4* expression and survival. These findings emphasize that *ATP2B4* expression in bulk colorectal tumor datasets should be interpreted within the cellular and molecular context of the tumor rather than solely as a tumor-cell-intrinsic characteristic.

The co-expression and functional enrichment analyses further demonstrated that *ATP2B4* expression was associated with distinct transcriptional programs in colorectal cancer. Genes positively correlated with *ATP2B4* were enriched in extracellular matrix organization, cell adhesion, focal adhesion, and integrin-related processes, whereas negatively associated gene sets included mitochondrial and oxidative phosphorylation-related processes. TNS1, a focal adhesion-associated protein involved in integrin–cytoskeletal interactions [28], showed a particularly strong positive correlation with *ATP2B4* (r = 0.8245). Notably, a recent multi-omics study in CRC identified a TNS1–FERMT2–FN1–integrin signaling axis, further linking TNS1 to integrin-associated transcriptional programs in CRC [29]. These associations are consistent with the relationship between *ATP2B4* expression and stromal content observed in GSE39582 and may therefore partly reflect differences in tumor cellular composition. Nevertheless, previous experimental evidence has shown that PMCA4 can participate in epithelial organization; PMCA4b has recently been reported to promote epithelial polarization and lumen formation through regulation of E-cadherin trafficking [30]. Thus, the observed associations between *ATP2B4* and adhesion-related transcriptional programs have a plausible biological context, although the present co-expression and enrichment analyses do not establish that PMCA4 directly regulates these pathways in colorectal cancer.

Several limitations should be considered when interpreting the present findings. First, this study was based on retrospective analyses of publicly available datasets, and differences in patient populations, analytical platforms, and data-processing procedures across cohorts may have contributed to variation in the observed associations. Second, gene-expression measurements from bulk tumor tissues represent mixtures of malignant epithelial, stromal, and immune cells. Although StromalScore, estimated tumor purity, and CMS were incorporated into the analyses, these approaches cannot directly determine the cellular source of *ATP2B4* expression. Third, co-expression and enrichment analyses identify transcriptional associations and do not establish causal or functional relationships between *ATP2B4* and the associated biological pathways. In addition, no experimental validation was performed in the present study. Nevertheless, the reproducibility of reduced *ATP2B4* expression was evaluated in an independent matched cohort, and the prognostic analyses were examined using an independent cohort, multiple *ATP2B4*-associated probes, multivariable models, and analyses accounting for tumor composition and molecular subtype. Future studies integrating single-cell or spatial transcriptomic approaches with functional experiments will be required to determine the cell-type-specific expression and biological roles of PMCA4 in colorectal cancer and to clarify whether its expression has clinical relevance in defined molecular or cellular contexts.

## 5. Conclusions

In conclusion, our integrative analyses demonstrate that *ATP2B4*/PMCA4 expression is consistently reduced in colorectal tumors across independent transcriptomic and proteomic datasets. Although higher *ATP2B4* expression was associated with poorer overall survival in the KM Plotter cohort, this association was not consistently reproduced in an independent cohort and was attenuated after accounting for tumor composition and molecular subtype. *ATP2B4* expression was also associated with stromal content, tumor purity, CMS, and distinct transcriptional programs related to extracellular matrix organization, cell adhesion, and mitochondrial processes. These findings highlight the importance of considering the cellular and molecular context when interpreting *ATP2B4* expression in bulk colorectal tumor datasets. Further studies using single-cell or spatial approaches together with functional experiments are required to determine the cell-type-specific expression and biological roles of PMCA4 and to clarify its potential clinical relevance in colorectal cancer.

## Figures and Tables

**Figure 1:**
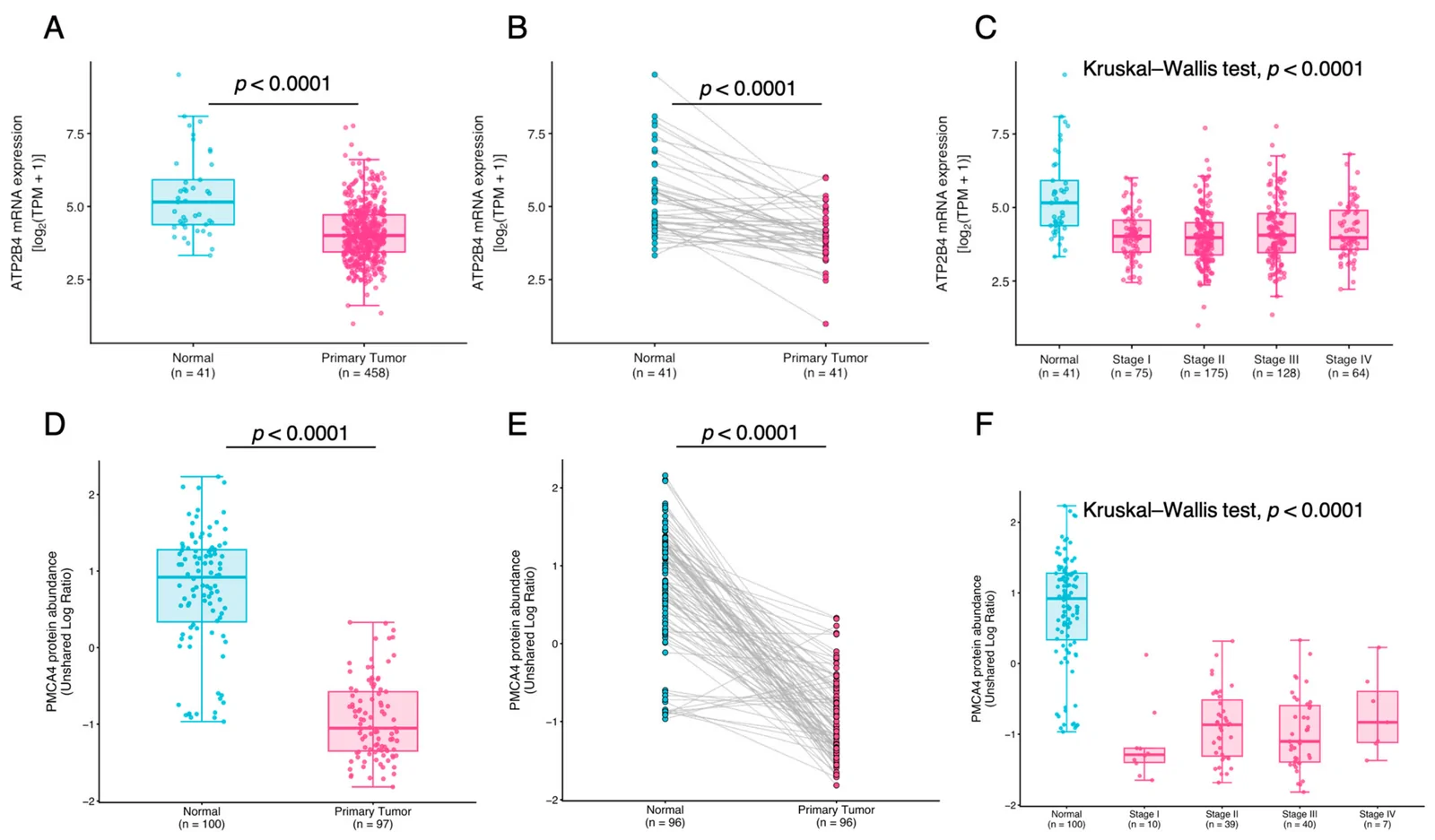
*ATP2B4* mRNA and PMCA4 protein expression in colorectal cancer. (**A**) *ATP2B4* mRNA expression in normal colon tissues (*n* = 41) and primary tumors (*n* = 458) in the TCGA-COAD cohort. (**B**) Paired comparison of *ATP2B4* mRNA expression between matched normal and primary tumor tissues (*n* = 41 pairs). (**C**) *ATP2B4* mRNA expression in normal tissues (*n* = 41) and primary tumors stratified by pathological stage (stage I, *n* = 75; stage II, *n* = 175; stage III, *n* = 128; stage IV, *n* = 64). *ATP2B4* expression is presented as log_2_(TPM + 1). (**D**) PMCA4 protein abundance in normal tissues (*n* = 100) and primary tumors (*n* = 97) in the CPTAC colon cancer cohort. (**E**) Paired comparison of PMCA4 protein abundance between matched normal and primary tumor tissues (*n* = 96 pairs). (**F**) PMCA4 protein abundance in normal tissues (*n* = 100) and primary tumors stratified by pathological stage (stage I, *n* = 10; stage II, *n* = 39; stage III, *n* = 40; stage IV, *n* = 7). Protein abundance is presented as Unshared Log Ratio. Unpaired normal–tumor comparisons were performed using the Wilcoxon rank-sum test, and paired comparisons were performed using the Wilcoxon signed-rank test. Differences among normal tissues and pathological stages were assessed using the Kruskal–Wallis test. Normal-versus-stage pairwise comparisons were performed using Wilcoxon rank-sum tests with Holm adjustment for multiple comparisons. Boxes indicate the interquartile range with the median shown by the center line, and whiskers extend to 1.5 times the interquartile range. Individual points represent individual samples. TCGA: The Cancer Genome Atlas; COAD: colon adenocarcinoma; CPTAC: Clinical Proteomic Tumor Analysis Consortium; TPM: transcripts per million.

**Figure 2:**
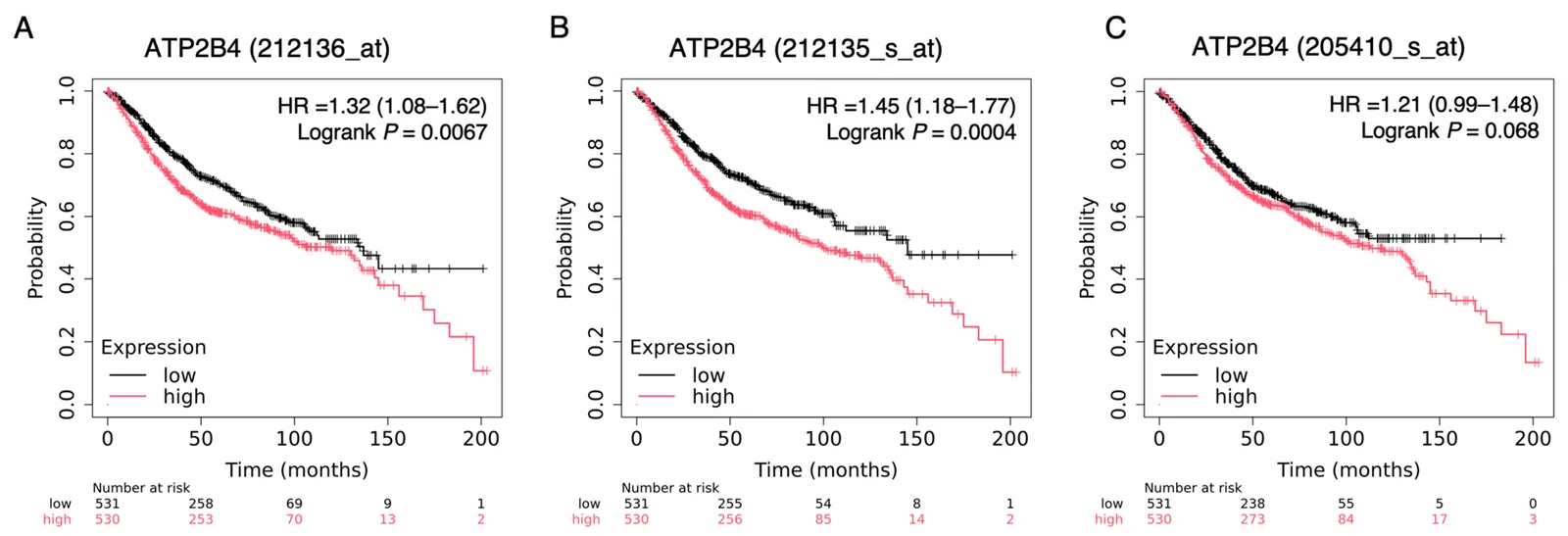
Prognostic associations of *ATP2B4*-associated microarray probes in colorectal cancer. Kaplan–Meier analysis of overall survival in patients with colorectal cancer (*n* = 1061) according to the expression of three *ATP2B4*-associated microarray probes in the KM Plotter dataset: (**A**) 212136_at, (**B**) 212135_s_at, and (**C**) 205410_s_at. Patients were dichotomized into high- and low-expression groups using the median expression of each probe (low, *n* = 531; high, *n* = 530). Hazard ratios (HRs) with 95% confidence intervals (CIs) and log-rank *p* values are shown in each panel. Numbers at risk are displayed below the Kaplan–Meier curves. In the KM Plotter database, probe 212136_at is annotated as MXRA1/*ATP2B4*.

**Figure 3:**
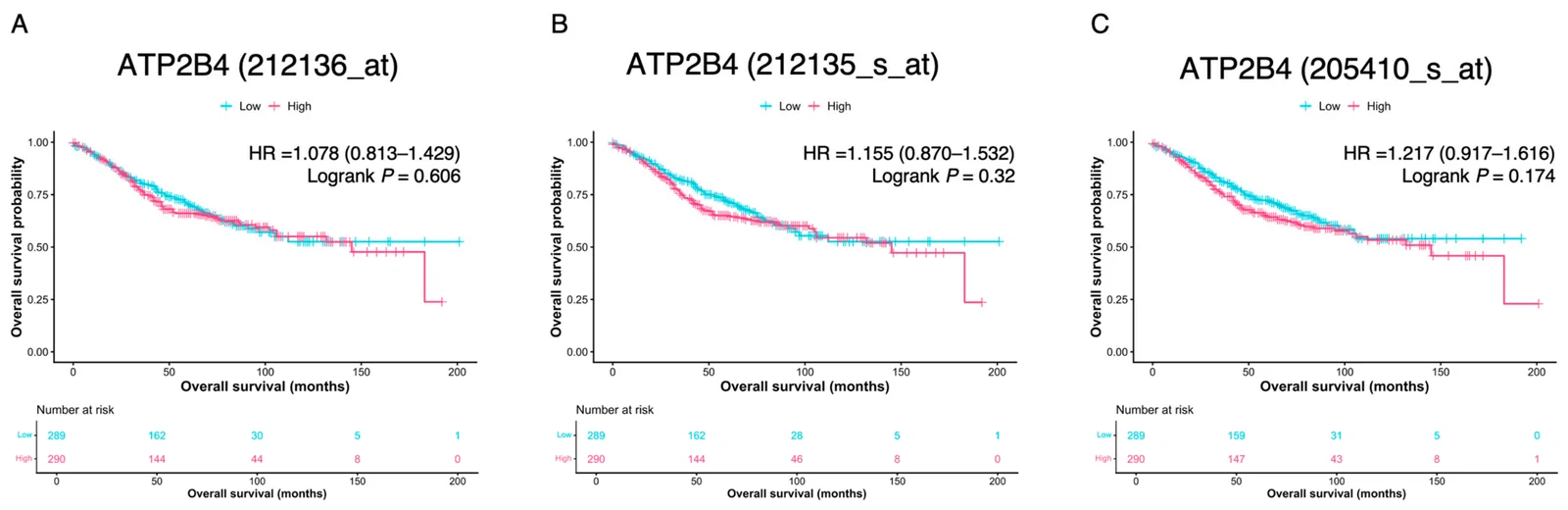
Independent validation of the prognostic associations of *ATP2B4*-associated probes in the GSE39582 cohort. Kaplan–Meier analysis of overall survival in patients with colorectal cancer from the GSE39582 cohort (*n* = 579; 194 events) according to the expression of three *ATP2B4*-associated microarray probes: (**A**) 212136_at, (**B**) 212135_s_at, and (**C**) 205410_s_at. Patients were dichotomized into low- and high-expression groups using the median expression of each probe (low, *n* = 289; high, *n* = 290). Expression values equal to the median were assigned to the high-expression group. Hazard ratios (HRs) comparing the high- with the low-expression group, 95% confidence intervals (CIs), and log-rank *p* values are shown in each panel. Numbers at risk are shown below the Kaplan–Meier curves.

**Figure 4:**
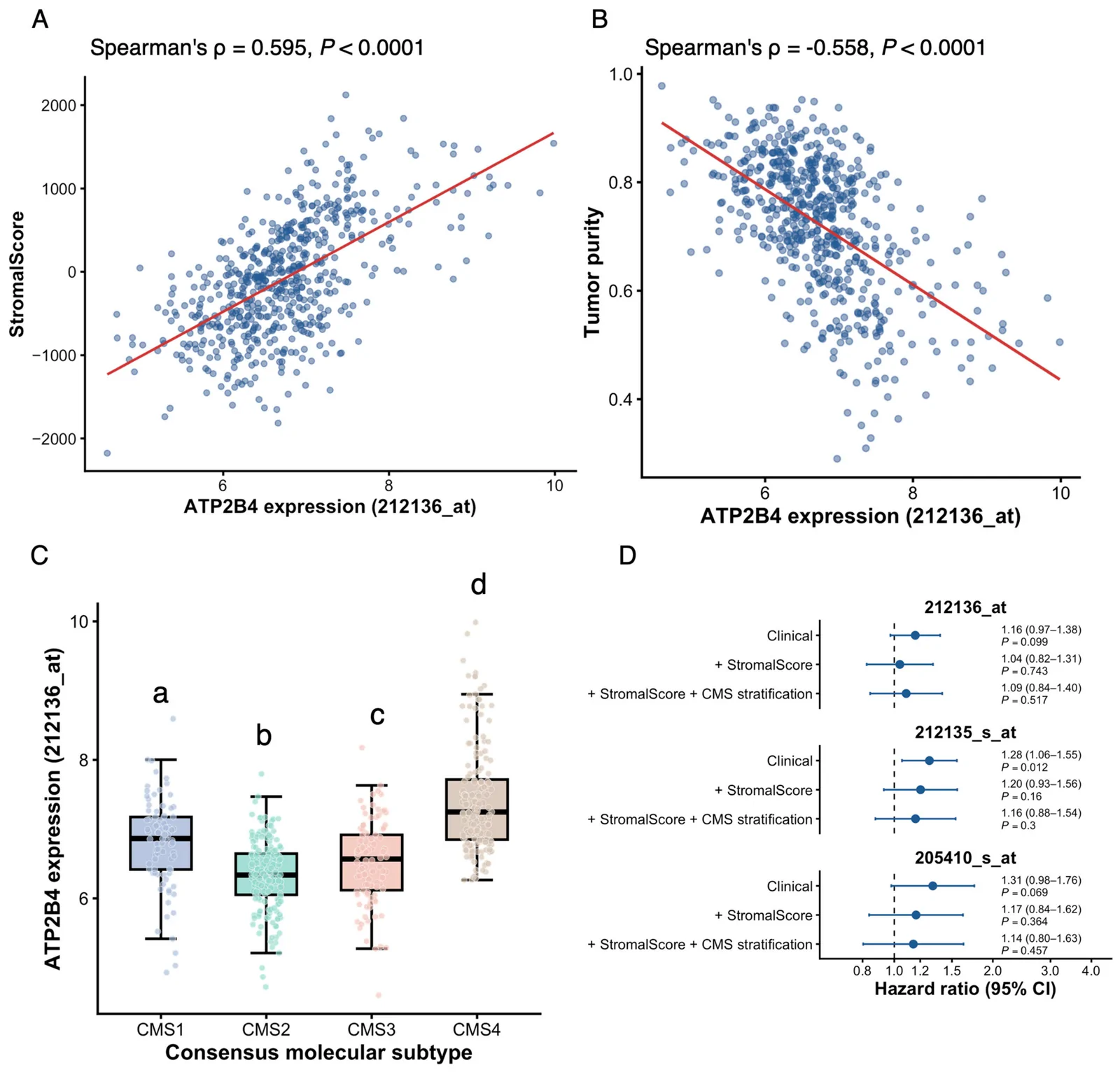
Association of *ATP2B4* expression with tumor composition and prognosis in the GSE39582 cohort. (**A**) Association between *ATP2B4* expression (212136_at) and ESTIMATE-derived StromalScore. (**B**) Association between *ATP2B4* expression (212136_at) and ESTIMATE-derived tumor purity. Associations in (**A**,**B**) were assessed using Spearman’s rank correlation; Spearman’s ρ and *p* values are shown in each panel. (**C**) *ATP2B4* expression (212136_at) across consensus molecular subtypes (CMS) (CMS1–CMS4; *n* = 521: CMS1, *n* = 83; CMS2, *n* = 176; CMS3, *n* = 98; CMS4, *n* = 164). Differences among CMS groups were assessed using the Kruskal–Wallis test, with the overall *p* value shown in the panel, followed by Dunn’s test with Benjamini–Hochberg adjustment for multiple comparisons. Boxes represent the interquartile range with the median indicated by the center line; whiskers extend to the most extreme values within 1.5 times the interquartile range. Individual points represent individual tumors. (**D**) Multivariable Cox proportional hazards analyses of overall survival for three *ATP2B4*-associated probes (212136_at, 212135_s_at, and 205410_s_at). The clinical model included *ATP2B4* expression, age, sex, and TNM stage; the second model additionally included StromalScore; and the final model additionally incorporated CMS as a stratification factor, allowing baseline hazard functions to differ among CMS1–CMS4. Hazard ratios (HRs) for *ATP2B4* probe expression are shown with 95% confidence intervals (CIs) and *p* values.

**Figure 5:**
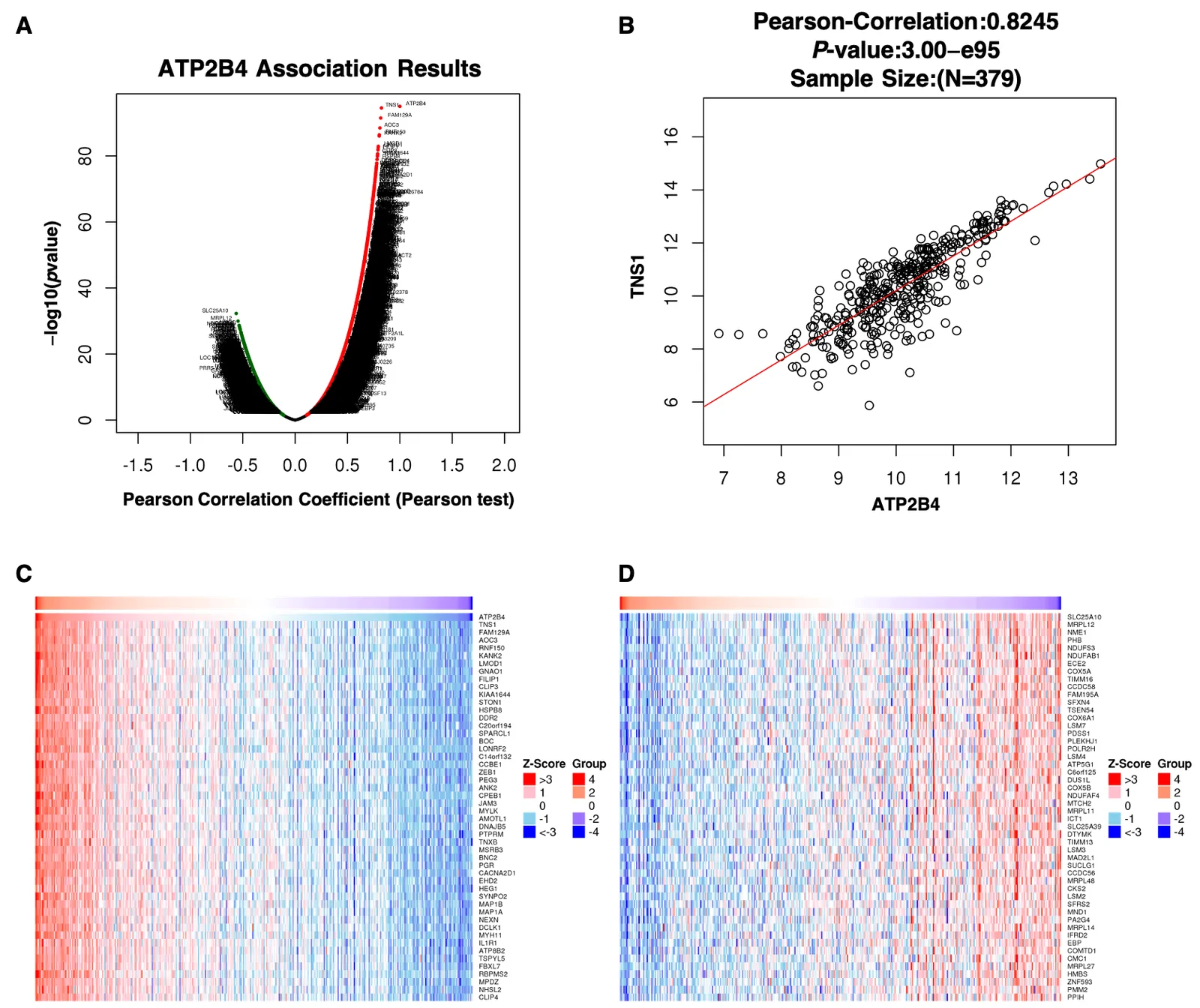
Co-expression analysis of *ATP2B4* in colorectal cancer. (**A**) Volcano plot showing genes positively and negatively correlated with *ATP2B4* expression in the TCGA COADREAD cohort (*n* = 379) using the LinkedOmics database. Positively and negatively correlated genes are shown in red and green, respectively. (**B**) Pearson correlation between *ATP2B4* and TNS1 expression. The red line indicates the linear regression line. (**C**) Heatmap of the top 50 genes positively correlated with *ATP2B4* expression. (**D**) Heatmap of the top 50 genes negatively correlated with *ATP2B4* expression. Pearson correlation analysis was performed using the LinkedOmics platform.

**Figure 6:**
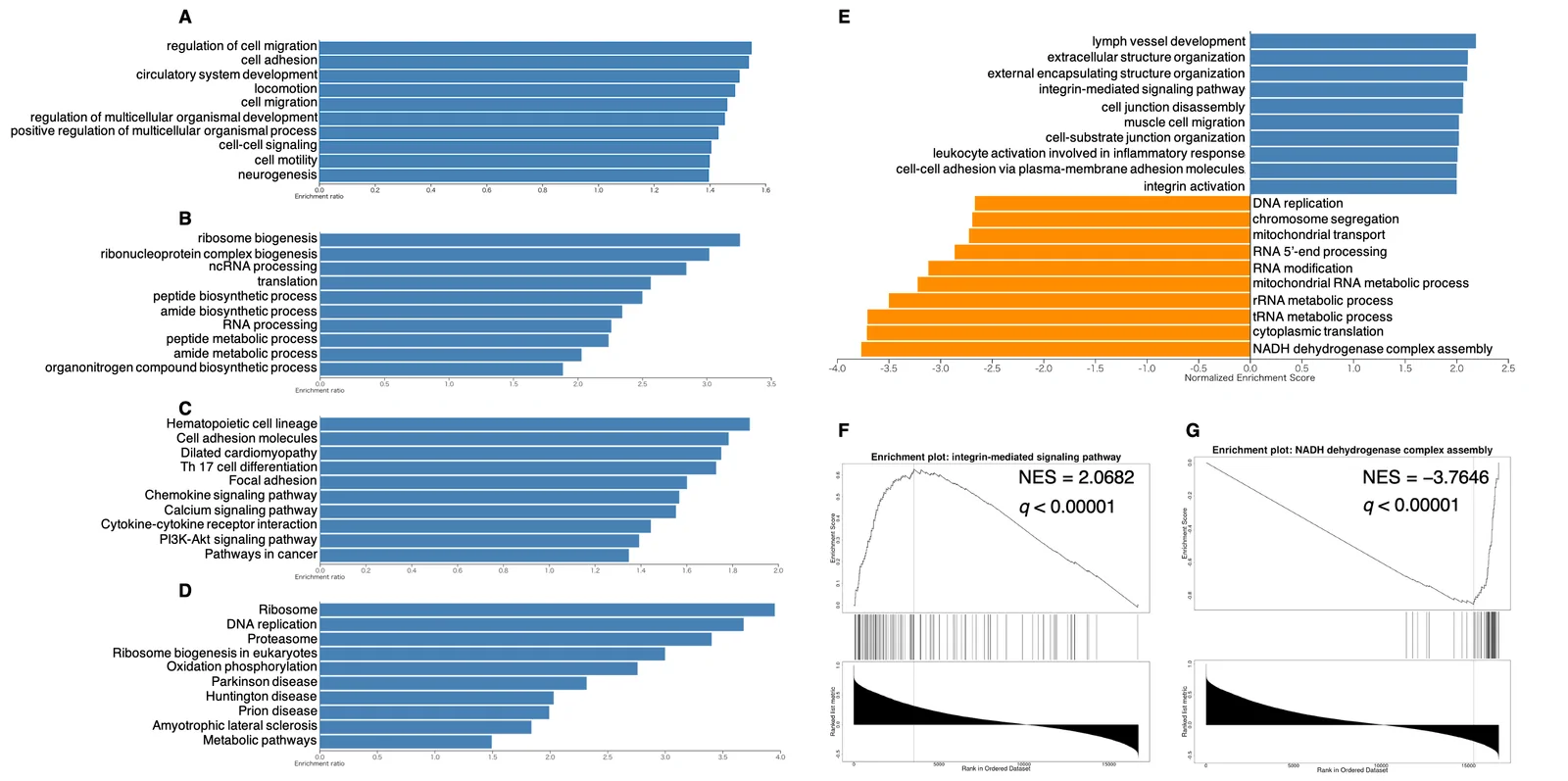
Functional enrichment analysis of *ATP2B4*-associated genes in colorectal cancer. (**A**) Gene Ontology (GO) biological process enrichment analysis of genes positively correlated with *ATP2B4* expression. (**B**) GO biological process enrichment analysis of genes negatively correlated with *ATP2B4* expression. (**C**) Kyoto Encyclopedia of Genes and Genomes (KEGG) pathway enrichment analysis of genes positively correlated with *ATP2B4* expression. (**D**) KEGG pathway enrichment analysis of genes negatively correlated with *ATP2B4* expression. (**E**) Gene set enrichment analysis (GSEA) showing gene sets positively and negatively associated with *ATP2B4* expression. (**F**) Representative GSEA enrichment plot for the integrin-mediated signaling pathway, which was positively associated with *ATP2B4* expression. (**G**) Representative GSEA enrichment plot for NADH dehydrogenase complex assembly, which was negatively associated with *ATP2B4* expression. Functional enrichment analyses were performed using the LinkedOmics platform. Only significantly enriched GO terms, KEGG pathways, and GSEA gene sets with a false discovery rate (FDR) < 0.05 are shown.

**Table 1:** Multivariable Cox proportional hazards analysis of *ATP2B4*-associated probe expression and overall survival in the GSE39582 cohort.

Probe	Model	N	Events	HR	95% CI	*p* Value
212136_at	Clinical model	574	193	1.16	0.97–1.38	0.099
212136_at	+ StromalScore	574	193	1.04	0.82–1.31	0.743
212136_at	+ StromalScore, stratified by CMS	510	171	1.09	0.84–1.40	0.517
212135_s_at	Clinical model	574	193	1.28	1.06–1.55	0.012
212135_s_at	+ StromalScore	574	193	1.2	0.93–1.56	0.16
212135_s_at	+ StromalScore, stratified by CMS	510	171	1.16	0.88–1.54	0.3
205410_s_at	Clinical model	574	193	1.31	0.98–1.76	0.069
205410_s_at	+ StromalScore	574	193	1.17	0.84–1.62	0.364
205410_s_at	+ StromalScore, stratified by CMS	510	171	1.14	0.80–1.63	0.457

The clinical model included probe expression, age, sex, and TNM stage. The second model additionally included StromalScore. In the final model, CMS was incorporated as a stratification factor in addition to the variables included in the second model, allowing baseline hazard functions to vary across CMS1–CMS4. HR: hazard ratio; CI: confidence interval; CMS: consensus molecular subtype.

## Data Availability

All data analyzed in this study are publicly available from the TCGA, CPTAC, GEO, KM Plotter, and LinkedOmics databases. The databases used in the initial analyses were first accessed on 15 July 2026. During revision, the relevant databases were re-accessed between 25 and 31 August 2026, and additional datasets were obtained for the revised and independent validation analyses. Detailed analysis procedures and parameters are described in the Methods section. Additional information is available from the corresponding author upon reasonable request.

## Supplementary Materials

The following supporting information can be downloaded at https://www.mdpi.com/article/10.3390/cimb48100977/s1.
