# Supplementary material for: Integrative Analysis of ATP2B4 Expression and Its Clinical and Molecular Associations in Colorectal Cancer
Source: Curr Issues Mol Biol. 2026 Sep 23;48(10):977. doi: 10.3390/cimb48100977 (PMC13703047; doi:10.3390/cimb48100977)
Supplement: Supplementary file 1 [file cimb-48-00977-s001.zip › cimb-4506461-supplementary files/cimb-4506461-supplementary figures and tables.pdf]

Integrative Analysis of ATP2B4 Expression and Its Clinical and Molecular Associations in Colorectal Cancer

Kai Aoki, Mitsugi Shimoda, Kazuhiro Takekoshi

Supplementary Figures and Tables

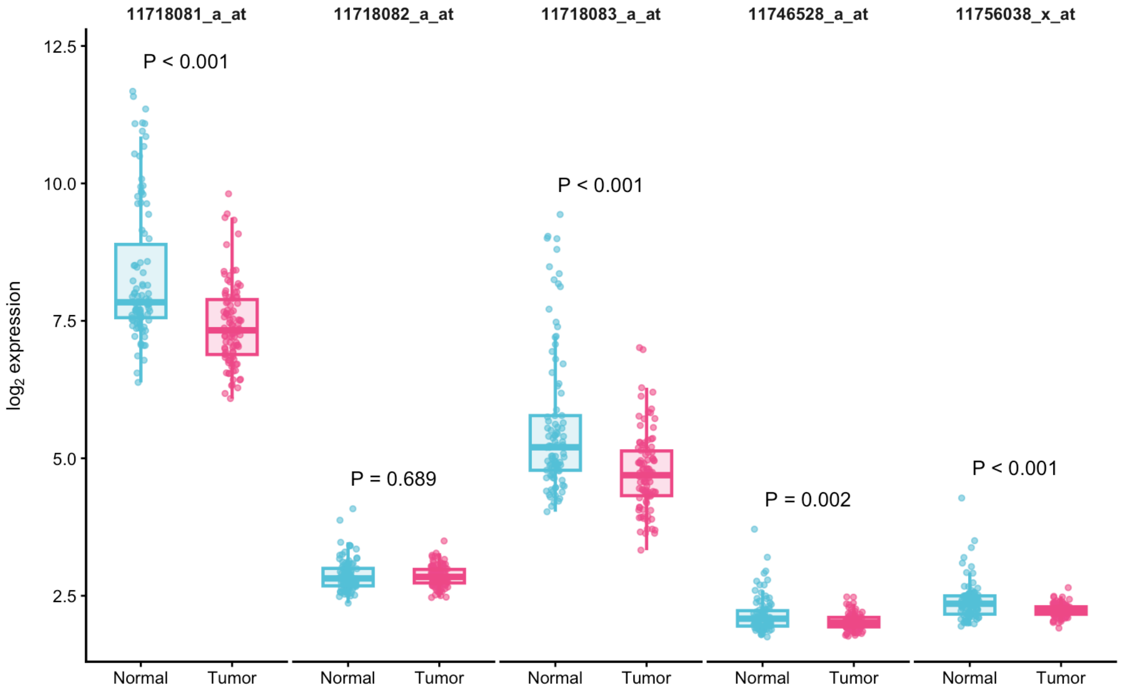

Figure S1. Validation of ATP2B4 expression in an independent colorectal cancer cohort. ATP2B4 expression was evaluated in the independent GSE44076 cohort using five ATP2B4-associated probes. Expression levels were compared between matched normal colorectal mucosa (n = 98) and primary colorectal tumor tissues (n = 98) from 98 patients (98 matched pairs). Boxplots show the median and interquartile range, with whiskers extending to the most extreme values within 1.5 times the interquartile range; individual points represent individual samples. Comparisons between matched normal and tumor tissues were performed using the paired Wilcoxon signed-rank test. Raw *P* values are shown for each probe.

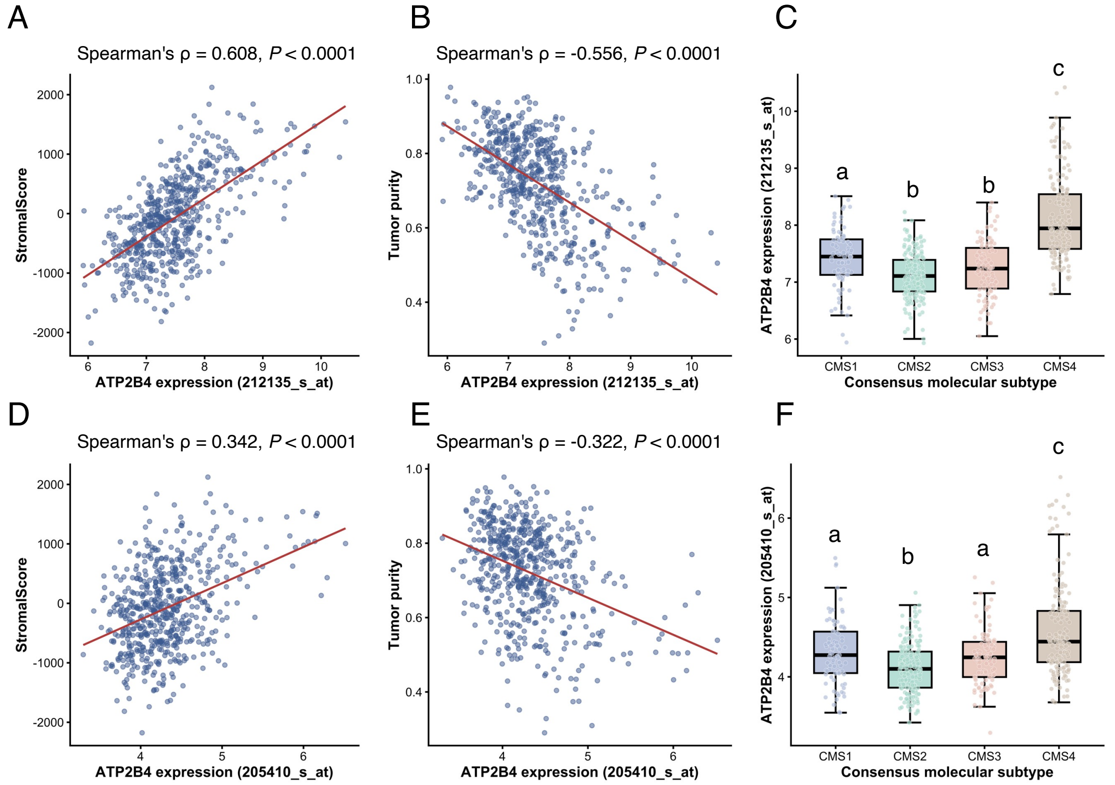

Figure S2. Associations of alternative ATP2B4-associated probes with tumor composition and consensus molecular subtype in the GSE39582 cohort.

(A–C) Analyses using the ATP2B4-associated probe 212135_s_at. (A) Association between probe expression and ESTIMATE-derived StromalScore. (B) Association between probe expression and estimated tumor purity. (C) Probe expression across consensus molecular subtypes (CMS1–CMS4). (D–F) Corresponding analyses using the alternative ATP2B4-associated probe 205410_s_at. (D) Association between probe expression and StromalScore. (E) Association between probe expression and estimated tumor purity. (F) Probe expression across CMS1–CMS4. Associations with StromalScore and tumor purity were assessed using Spearman's rank correlation; red lines indicate linear regression fits for visualization. Differences among CMS groups were assessed using the Kruskal–Wallis test followed by Dunn's post hoc test with Benjamini–Hochberg adjustment for multiple comparisons. Different lowercase letters indicate statistically significant differences between CMS groups based on the adjusted pairwise comparisons (adjusted $P < 0.05$); groups sharing the same letter were not significantly different. CMS classification was available for 521 tumors (CMS1, n = 83; CMS2, n = 176; CMS3, n = 98; and CMS4, n = 164). Boxes represent the interquartile range with the median indicated by the center line; whiskers extend to the most extreme values within 1.5 times the interquartile range, and individual points represent individual tumor samples.

Table S1. Pairwise comparisons of ATP2B4 mRNA and PMCA4 protein abundance between normal tissues and pathological stages in TCGA-COAD and CPTAC cohorts

Cohort	Comparison	Normal, n	Tumor, n	Raw *P* value	Holm-adjusted *P* value
TCGA-COAD	Normal vs. Stage I	41	75	1.96×10^{-7}	5.87×10^{-7}
	Normal vs. Stage II	41	175	2.27×10^{-9}	9.08×10^{-9}
	Normal vs. Stage III	41	128	2.75×10^{-6}	5.49×10^{-6}
	Normal vs. Stage IV	41	64	1.52×10^{-5}	1.52×10^{-5}
CPTAC	Normal vs. Stage I	100	10	9.47×10^{-7}	1.89×10^{-6}
	Normal vs. Stage II	100	39	1.11×10^{-15}	3.33×10^{-15}
	Normal vs. Stage III	100	40	$<1 \times 10^{-15}$	$<1 \times 10^{-15}$
	Normal vs. Stage IV	100	7	2.18×10^{-4}	2.18×10^{-4}

Pairwise comparisons between normal tissues and each pathological stage were performed using the Wilcoxon rank-sum test. P values were adjusted for the four pairwise comparisons within each cohort using the Holm method. TCGA-COAD analyses were based on ATP2B4 mRNA expression, whereas CPTAC analyses were based on PMCA4 protein abundance.

Table S2. Comparison of ATP2B4-associated probe expression between matched normal colorectal mucosa and primary colorectal tumors in the GSE44076 cohort

Probe	Matched pairs, n	Mean normal	Mean tumor	Mean difference	Raw *P* value	BH-adjusted *P* value
11718081_a_at	98	8.325	7.425	–0.900	6.39×10^{-8}	1.98×10^{-7}
11718082_a_at	98	2.870	2.855	–0.015	0.689	0.689
11718083_a_at	98	5.607	4.750	–0.857	7.94×10^{-8}	1.98×10^{-7}
11746528_a_at	98	2.150	2.025	–0.125	1.52×10^{-3}	1.90×10^{-3}
11756038_x_at	98	2.394	2.233	–0.161	2.40×10^{-5}	4.00×10^{-5}

ATP2B4 expression was evaluated using five ATP2B4-associated probes in matched normal colorectal mucosa and primary colorectal tumor tissues from 98 patients (98 matched pairs). Differences between matched normal and tumor tissues were assessed using paired Wilcoxon signed-rank tests. Raw P values were adjusted across the five probes using the Benjamini–Hochberg method. BH-adjusted P values < 0.05 were considered statistically significant.

Table S3. Pairwise comparisons of ATP2B4-associated probe expression among consensus molecular subtypes in the GSE39582 cohort

Probe	Comparison	Raw *P* value	BH-adjusted *P* value
212136_at	CMS1 vs. CMS2	7.05×10^{-9}	1.41×10^{-8}
	CMS1 vs. CMS3	0.0029	0.0035
	CMS2 vs. CMS3	0.0095	0.0095
	CMS1 vs. CMS4	5.10×10^{-7}	7.65×10^{-7}
	CMS2 vs. CMS4	1.42×10^{-40}	8.49×10^{-40}
	CMS3 vs. CMS4	1.67×10^{-18}	5.01×10^{-18}
212135_s_at	CMS1 vs. CMS2	1.72×10^{-5}	2.58×10^{-5}
	CMS1 vs. CMS3	0.0161	0.0193
	CMS2 vs. CMS3	0.0904	0.0904
	CMS1 vs. CMS4	8.58×10^{-11}	1.72×10^{-10}
	CMS2 vs. CMS4	1.57×10^{-40}	9.43×10^{-40}
	CMS3 vs. CMS4	4.52×10^{-22}	1.35×10^{-21}
205410_s_at	CMS1 vs. CMS2	1.10×10^{-4}	2.20×10^{-4}
	CMS1 vs. CMS3	0.3957	0.3957
	CMS2 vs. CMS3	0.0021	0.0031
	CMS1 vs. CMS4	0.0022	0.0026
	CMS2 vs. CMS4	1.26×10^{-17}	7.58×10^{-17}
	CMS3 vs. CMS4	2.39×10^{-5}	7.18×10^{-5}

Consensus molecular subtype (CMS) classification was available for 521 tumors in the GSE39582 cohort (CMS1, n = 83; CMS2, n = 176; CMS3, n = 98; and CMS4, n = 164). Overall differences in probe expression among CMS1–CMS4 were assessed using the Kruskal–Wallis test, followed by Dunn's post hoc test for pairwise comparisons. P values from Dunn's tests were adjusted for multiple comparisons using the Benjamini–Hochberg method. BH-adjusted P values < 0.05 were considered statistically significant.
